# Retraction: ILC2s improve glucose metabolism through the control of saturated fatty acid absorption within visceral fat

**DOI:** 10.3389/fimmu.2026.1937276

**Published:** 2026-07-17

**Authors:** Frontiers Editorial Office

The journal retract the cited article above.

Following publication, concerns were raised regarding the integrity of the images and data in the published article. The authors failed to provide a satisfactory explanation during the investigation, which was conducted in accordance with Frontiers’ policies.

This retraction was approved by the Chief Editors of Frontiers in Immunology and the Chief Executive Editor of Frontiers. The authors received a communication regarding the retraction and had a chance to respond. This communication has been recorded by the publisher.

